# Should Pyrexia Be Treated?

**Published:** 1915-11-06

**Authors:** 


### SHOULD PYREXIA BE TREATED?

The clinical thermometer is a very useful instrument, or even an essential one, but its records are sometimes regarded with exaggerated reverence. There are some domestic circles where it is sedulously cultivated and where the inhabitants of the nursery, and even more mature organisations, are made to suffer it as a daily discipline. Here the slightest departure from what is marked as a sacred standard excites alarm and is apt to lead to remedial intervention. Again, during the course of a febrile illness the patient, and still more his friends, often wait upon the movements of the mercury as affording a certain index, one way or the other, of the progress of the malady, and hopes rise or fall inversely to the temperature reading. All this, of course, means an exaggerated estimate of the value and meaning of such readings, and it leads to a kind of popular pressure that direct attempts should be made to beat down the temperature whenever this can be accused of passing above the stated normal limit.

Assuming, however, the existence of pyrexia as determined by a competent observer, the question arises whether any attempts to treat the symptom as such should be made. There is one condition—namely, hyperpyrexia—in which the answer to this question would be almost unanimously in the affirmative. It is allowed that here the temperature reaches a level at which it may be directly prejudicial or even dangerous to life. From such a conclusion it is not unnatural to proceed to the opinion that lesser degrees of fever must also be hurtful, and that when they exist there is a therapeutic indication for the employment of vigorous anti-pyretic measures. In this way there arose the energetic use of cold baths and of various drugs whenever the temperature reached a distinctly febrile level. The fever in itself was regarded as a mischievous thing, and there was therefore a claim to abate it by artificial means. To this doctrine and practice there succeeded a reaction based partly upon observation, partly on hypothesis. The opinion was ventured that a definitely raised temperature level was the sign of a vigorous tissue reaction against the microbic causes of the disease; or, again, that the mere lowering of the temperature did not touch the conditions of which the pyrexia was but the expression. Then, as a matter of actual experience, it became manifest that at least some of the agents which depressed the temperature depressed also other functions, and that the gain—allowing it to be so—in one direction was more than neutralised by loss in others. Under these influences anti-pyretic practice, as a matter of routine at least, lost its hold on professional judgment and was very largely abandoned.

It is reasonable to ask whether the reaction against the use of anti-pyretics may not have gone too far. Admitting that at one time there was excess, and that in short-lived febrile disturbances there is no need for these drugs, it may still be possible that there is a field for them in more prolonged febrile states. That we cannot cure the disease is no reason that we should not relieve a symptom, provided that this result can be achieved without inflicting harm in other directions. Further, whilst a moderately high temperature in an acute process may be negligible, or even advantageous, it is another matter when this occurs as a daily experience over a matter of weeks and months. Inevitably such a succession produces certain consequences which are disadvantageous to the patient, and some of these are a direct outcome of the abnormal temperature. If this latter may be removed or qualified, the evils which follow from it will be lessened or avoided. Particularly is there a field for the application of this principle in cases where pyrexia appears daily at a certain fixed hour. Here by suitable remedies and the careful adjustment of the time of administration the threatened rise of the thermometer may be anticipated and defeated, with distinct advantage to the patient. Hence, while admitting freely that the practice of smiting indiscriminately at all febrile records is absurd and unwise, we think there is a distinct opportunity for the use of anti-pyretic drugs, and particularly in the circumstances we have just defined.

